# Risk Trajectories of Type 2 Diabetes–Related Microvascular Complications Across Racial/Ethnic Groups Among Older Adults

**DOI:** 10.1007/s40615-025-02625-0

**Published:** 2025-09-09

**Authors:** Kaustubh V. Parab, Xiaotian Gao, Neha P. Gothe, Kenneth R. Wilund, Harshal P. Mahajan, Chelsea R. Singleton

**Affiliations:** 1 Department of Cardiovascular and Thoracic Services, Aurora St. Luke’s Medical Center, Milwaukee, WI USA; 2 Center for Epidemiology and Population Health, Pediatrics, Baylor College of Medicine, Houston, TX USA; 3 Department of Physical Therapy, Human Movement, and Rehabilitation Sciences, Northeastern University, Boston, MA USA; 4 School of Nutritional Sciences and Wellness, University of Arizona, Tucson, AZ USA; 5 Department of Health and Kinesiology, College of Applied Health Sciences, University of Illinois Urbana-Champaign, Champaign, IL USA; 6 Department of Social, Behavioral, and Population Sciences, Tulane School of Public Health & Tropical Medicine, New Orleans, LA USA

**Keywords:** Type 2 diabetes, Microvascular complications, Race/ethnicity, Disparity, Neuropathy, Nephropathy

## Abstract

**Introduction:**

Type 2 diabetes mellitus (T2DM) microvascular complications are a major public health issue that disproportionately affects racial/ethnic minorities in the US. We aimed to address the limited understanding of racial/ethnic disparities in the longitudinal natural history of microvascular complications over eight years among older adults with T2DM in the US and Canada.

**Methods:**

From 10,251 participants in the Action to Control Cardiovascular Risk in Diabetes (ACCORD) (2003–2009) trial, we derived 6323 participants. We examined racial/ethnic disparities of composite microvascular complications, nephropathy, neuropathy, and retinopathy outcomes among adults of Black (*n* = 1099), White (*n* = 4071), and Other races (*n* = 1156), over 8 years. We used linear mixed-effects model with random intercept to account for intra-person correlation.

**Results:**

Our linear mixed-effects models displayed that the odds of composite microvascular complications for other race, which primarily consisted of Hispanic adults declined over a period of time [OR: 0.95 (0.93–0.99)]. The odds increased over time for Black [1.06 (1.03–1.09)] and White [1.11 (1.09–1.13)] adults (*P* < 0.001).

**Conclusion:**

Other race adults had a declining risk of microvascular complications over time. This decline was not observed among Black and White adults.

**Supplementary Information:**

The online version contains supplementary material available at https://doi.org/10.1007/s40615-025-02625-0.

## Introduction

Development of microvascular complications is a common burden for persons living with type 2 diabetes mellitus (T2DM) [1]. Microvascular complications are complex disorders mainly linked with biological pathway originating with chronic hyperglycemia [2]. In the United States (US), recent evidence suggests that the prevalence for T2DM-related microvascular complications (e.g., nephropathy) is increasing more than the macrovascular complications (e.g., stable angina) among adults living with T2DM [3]. Within microvascular complications, prevalence of nephropathy is most common followed by neuropathy and lastly retinopathy [3]. Globally, microvascular complications have resulted in increased risk of morbidity and mortality in T2DM patients and lower quality of life [1, 4].

The rising prevalence of microvascular complications among adults with T2DM is concerning given the documented disparities. Both US and non-US-based studies have reported that racial/ethnic minorities experience higher risk of T2DM microvascular complications than White adults [3, 5–8]. For example, a study with more than 62,000 T2DM patients in the US found higher incidence of nephropathy among Black, Hispanic, and Asian patients compared to White patients [5]. On the contrary, some studies found a lower prevalence of T2DM microvascular complications among racial/ethnic minorities in comparison to non-minority populations [3, 6, 9]. Among these studies, only one, a California-based cohort study, examined a US-based population [3]. This study found that White adults had the highest cumulative incidence of renal complication and all-cause mortality among T2DM patients. Because of the ambiguous findings across studies, research is needed to strengthen the evidence base by thoroughly evaluating racial/ethnic disparities in a large and diverse US and Canada-based population.

No prior multicenter longitudinal study based on Action to Control Cardiovascular Risk in Diabetes (ACCORD) data has simultaneously examined racial/ethnic risk trajectories of nephropathy, neuropathy, and retinopathy in older adults across multiple racial/ethnic groups. Laiteerapong et al. and An et al. were among the few studies to analyze data longitudinally [3, 10]. However, the sample for both the studies was based on a single site located in Southern or Northern California. Additionally, both studies evaluated outcomes that were diagnosed clinically. Therefore, clinically undiagnosed outcomes, which tend to be more prevalent in resource-limited racial/ethnic minorities, remained unaccounted [5, 8]. Among the recent US-based T2DM trials, ACCORD and Diabetes Prevention Program Outcome Study (DPPOS) measured microvascular complications objectively to account for both the diagnosed and undiagnosed complications longitudinally. The DPPOS study found the aggregated microvascular outcome similar across racial/ethnic groups. However, An ongoing DPPOS study, till 2014, recorded microvascular complications only in the 11th study year. As a result, the DPPOS may have missed the course of the natural history of microvascular complications progressing each year. Based on ACCORD data, Gerber et al. reported no difference in chronic kidney disease by race; however, other microvascular complications were not studied in detailed [11]. Our research addresses these limitations of similar studies by examining a multicenter, US and Canada-based racial/ethnic population with objectively measured microvascular complications each year longitudinally.

It is essential to study racial/ethnic disparities within the risk of microvascular outcomes longitudinally because recent studies demonstrated that the onset and progression of T2DM occurred earlier among Black adults than other racial/ethnic groups [9, 12]. Furthermore, T2DM among Black adults worsened over two years while other races either improved or remained stable [12]. These studies suggest that there may be structural factors, such as limited access to diabetes management resources, which may contribute to racial/ethnic disparities in T2DM risk and risk of microvascular complications [13–15]. Overall, this highlights the need to further study risk trajectories of microvascular complications by race/ethnicity, thereby identifying populations at risk that would benefit from targeted preventative and management interventions.

Based on the gaps in knowledge we have identified, there was a need to study racial/ethnic disparities in diagnosed and undiagnosed microvascular complications longitudinally. Thus, the objective of this study was to evaluate the risk of microvascular complications over years across racial/ethnic groups in the US (White, Black, and other race/ethnicity group including Hispanic/Latino, American Indian/Alaska Native, First Nation (Aboriginal Canadian), Asian, Native Hawaiian/Other, Pacific Islander, and French Canadian). Findings from this research will shed light on trajectories of microvascular complication risk across racial/ethnic groups, which may guide public health practitioners, educators, and clinicians seeking to develop targeted preventive measures. We hypothesized that there will be racial/ethnic differences in risk of composite microvascular complications, nephropathy, neuropathy, and retinopathy at baseline and as the disease progresses over years.

## Subjects, Materials, and Methods

### Data Source and Study Participants

The data source used for this secondary analysis was from the ACCORD study. The ACCORD trial (2003–2009) was conducted in seven clinical networks with 77 clinical sites within the US and Canada [16]. ACCORD was a double 2 × 2 factorial, parallel designed trial where all randomized participants received one of the two glycemic interventions (intensive vs. standard glycemia). Patients in both glycemic arms received oral anti-hyperglycemic medications and/or Insulin with advice on diet/lifestyle on an “as needed” basis to achieve target glycated hemoglobin level < 6% in intensive And 7.0–7.9% in the standard arm. Furthermore, 42% received one of the two blood pressure (BP) interventions (intensive vs standard BP), And 58% received one of the two lipid interventions (simvastatin with fibrate vs. with placebo). Intensive and standard BP groups received interventions to achieve different systolic BP goals (intensive: < 120 vs. standard: < 140 mm Hg). Thus, all participants were assigned to one of the eight ACCORD intervention groups. The ACCORD study was sponsored by National Institute of Health’s National Heart Lung Blood Institute And co-sponsored by National Institute of Diabetes And Digestive and Kidney Disease and Center for Disease Control and Prevention. A total of 10,251 trial participants were included because they met the criteria of having T2DM, HbA1c ≥ 7.5, aged 40–79 years with a history of cardiovascular diseases or 55–79 years with at least two risk factors for cardiovascular diseases. The loss to follow up was minimal, i.e., approximately 0.005% in both the intensive group and standard glycemic group. Intensive therapy was stopped due to increased mortality and patients were transferred into standard therapy group [17].

**Fig. 1:**
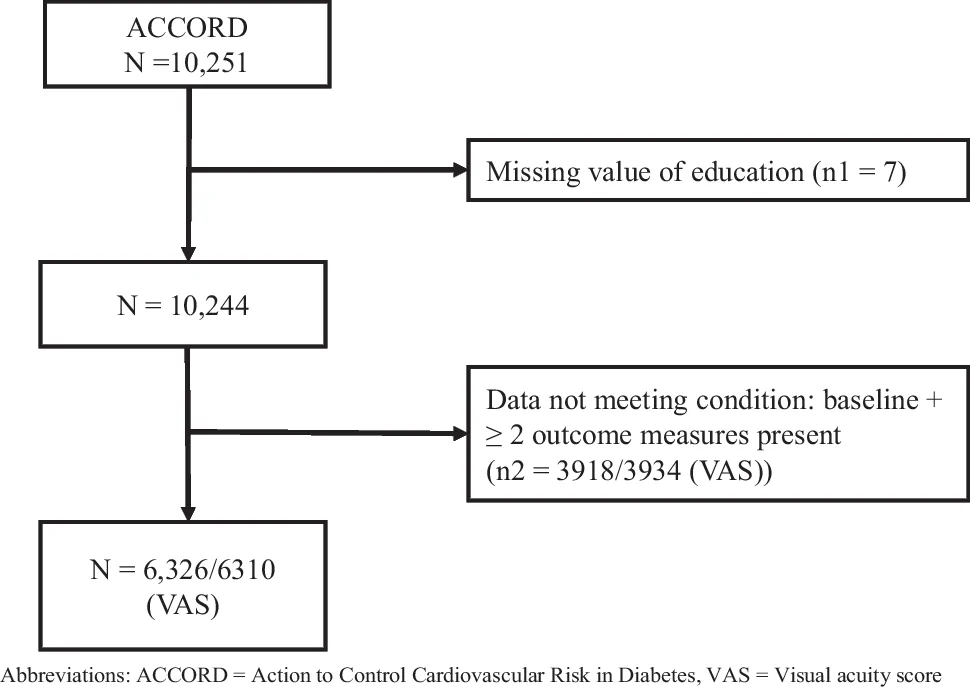
Study sample, ACCORD data (2003–2009; US and Canada; *N* = 6326). Abbreviations: ACCORD = Action to Control Cardiovascular Risk in Diabetes, VAS = Visual acuity score

For the current study, we excluded seven individuals without data on education level (Fig. 1). Participants were included if they had outcome readings for baseline and ≥ 2 years. We analyzed time points and removed years 6 and 7 due to lack of data provided. Similarly, for the visual acuity score (VAS) outcome, we removed years 1, 3, 5, 6, and 7 and kept only the baseline, years 2 and 4, and exit year. Timepoints were removed due to missingness and insufficient subsample for outcome measures in those years arising from uncertainty on data recording. For instance, VAS and severe vision loss had lower incidence of events and uncertainty due to merging category “not ascertained” with “no event” as per the original ACCORD study notes [16]. Thus, the total sample analyzed was 6326 participants. Each ACCORD trial site received Institutional Review Board approval, and all participants provided signed informed consent. The University of Illinois at Urbana-Champaign Institutional Review Board deemed this secondary analysis of the ACCORD study as exempt research.

### Microvascular Complications

Microvascular complications of interest included nephropathy, peripheral neuropathy, and retinopathy. All the baseline and yearly measurements were conducted by clinical coordinators at each clinical site and recorded at ACCORD clinics. For this study, we created composite and individual microvascular outcome variables with standard cutoff values to resemble ACCORD microvascular outcomes and standard definitions (Table 1). The composite microvascular outcome contained nephropathy based on serum creatinine (> 3.3 mg/dL), retinopathy based on vision loss, and neuropathy based on Michigan Neuropathy Screening Instrument (MNSI) score (> 2). Composite variables were included in the analysis to facilitate comparison with the primary ACCORD and UKPDS study measurements and reduce measurement bias. Nephropathy was measured using serum creatinine (mg/dL), estimated glomerular filtration rate (eGFR) from four-variable Modification of Diet in Renal Disease (MDRD) equation (ml/min/1.73 m^2^), and urine albumin to creatinine ratio (mg/g). Neuropathy was measured via standardized examinations such as MNSI score > 2, testing of vibratory sensation using 128 Hz tuning fork and Ankle jerk during Jendrassic maneuver, and light touch measured by 10 gm force monofilament test. Retinopathy was measured by visual acuity score based on Early Treatment of Diabetic Retinopathy Study (ETDRS ≤ 70), vision loss as measured by Snellen fraction < 20/200, and history of retinopathy. Other retinopathy measurements, including photocoagulation/vitrectomy treatment and cataract eye surgery, were not used in this analysis due to smaller subgroup samples.

**Table 1:** Definitions of microvascular outcomes we derived based on primary ACCORD study (2003–2009; US and Canada; *N* = 6326)

Outcome	Measurements
Composite microvascular complications	Serum creatinine (SCr) > 3.3 mg/dL or Michigan neuropathy screening instrument (MNSI) score > 2.0 or severe vision loss (SF < 20/200)
Composite nephropathy	Macro-albuminuria (UACR ≥ 300 mg/g) or SCr > 3.3 mg/dL or SCr doubling (≥ 2 times baseline) or ≥ 20 ml/min/1.73 m^2^ decrease in estimate glomerular filtration rate (eGFR)
Composite neuropathy	Presence/absence of MNSI > 2.0 or vibration perception at great toe or Ankle reflexes, or sensations from 10 g filament
Composite retinopathy	≤ 70 ETDRS or severe vision loss (SF < 20/200) or h/o retinopathy
Vision loss	Severe vision loss (SF < 20/200)

*ETDRS* Early Treatment Diabetic Retinopathy Study

### Other Variables

Participants self-reported their age (years), race/ethnicity, sex (male or female), diabetes years, education level, and alcohol intake at baseline. Race/ethnicity was reported as Black, White, Hispanic, and Other. In this study, we combined the Hispanic (*n* = 405) and other (*n* = 751) groups into a newly created other racial/ethnic group due to the lower sample size of participants in both groups. The diabetes years was recorded as type 2 diabetes duration in years. For modeling purposes, education levels were collapsed from four to the following two categories: ≤ high school graduate (or GED) and ≥ some college or technical school. Alcohol intake was measured as the number of alcoholic drinks consumed weekly. We collapsed eight categories of original ACCORD intervention arms to the following four arms: standard glycemia/BP trial, intensive glycemia/BP trial, standard glycemia/lipid trial, and intensive glycemia/lipid trial.

### Statistical Analysis

**Fig. 2:**
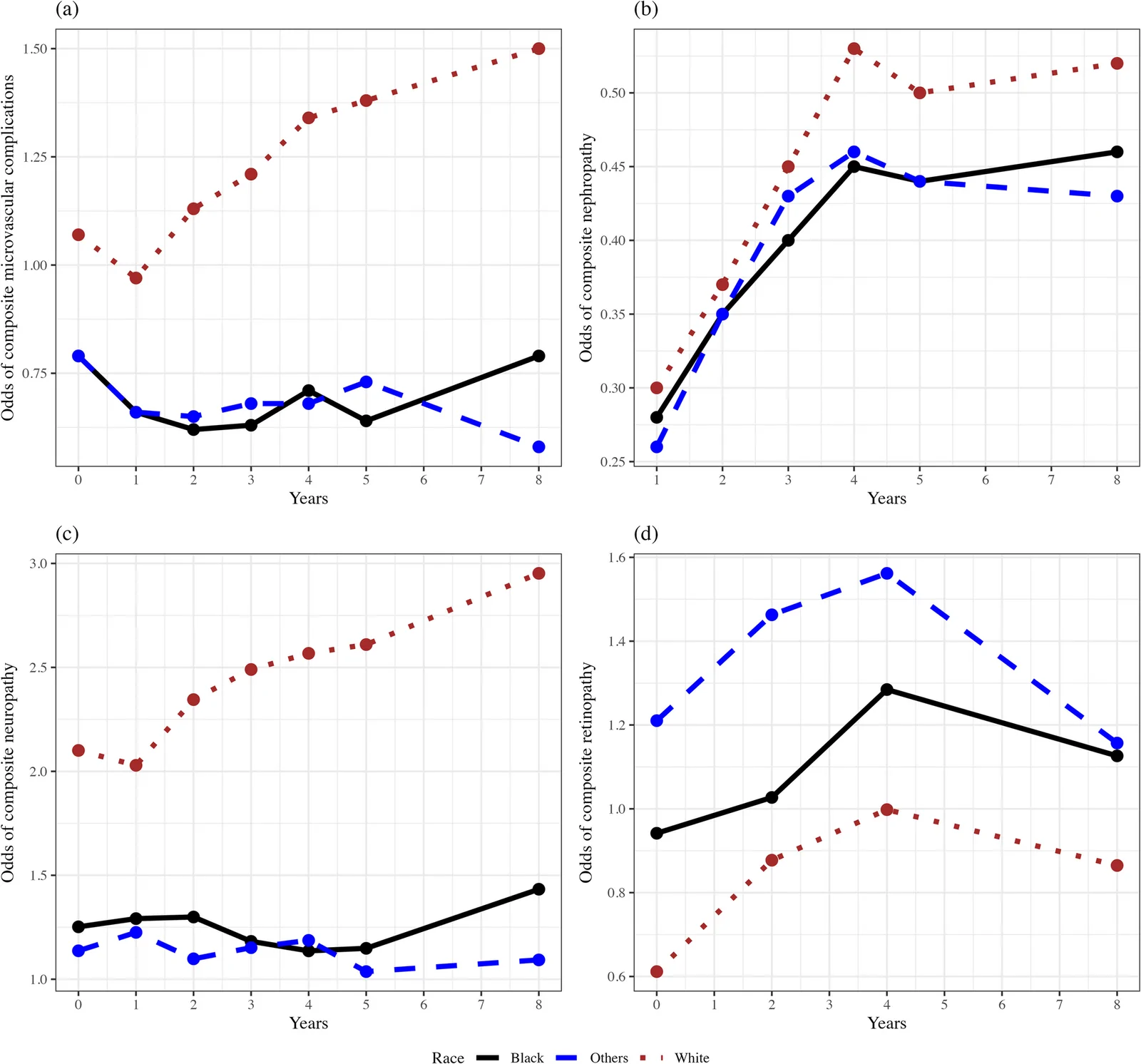
Unadjusted odds of microvascular complications. **a** composite microvascular complications, **b** composite nephropathy, **c** composite neuropathy, and **d** composite retinopathy by race/ethnicity over 8 years of follow-up, ACCORD data (2003–2009; US and Canada; *N* = 6326)

We compared baseline characteristics across race/ethnicity. We performed *χ*^2^ test and Fisher’s exact test for categorical variables, Kruskal–Wallis rank sum test (non-normally distributed) for continuous variables to determine the differences in measures by race/ethnicity, with *p* < 0.05 considered significant. We plotted unadjusted odds of microvascular complications by race/ethnicity groups over a period of 8 years. We also highlighted key time points when the outcomes were measured in our study (Fig. 2).

We used generalized linear mixed effects model with random intercept to account for intra-person correlation [18]. Random slope was not significant and therefore dropped. For each outcome (Table 1) stratified by racial/ethnicity groups, we included the baseline outcome, time (in years), sex, diabetes years, education, alcohol intake, and ACCORD intervention arms as fixed effects. Effect size in odds ratio of intercept and slope from such models was reported in Table 3. To test whether intercept and slopes were different across race/ethnicity groups, we fit the same mixed effects model to the entire population but additionally including interaction terms between race and intercept and between race and time. We then reported the overall Wald test *p* values for the interactions in Table 3. To specify the significantly differing racial/ethnic groups over years via linear contrasts, alpha < 0.02 was used. All analyses were conducted using R studio version 2023.12.1.402 and Stata version 18.0.

## Results

Table 2 displays descriptive statistics of our study sample from ACCORD data. We found significant racial/ethnic differences in terms of age, diabetes years, alcohol consumption, sex, education, and ACCORD intervention arms. In our study sample, White adults were relatively older, consumed more alcohol drinks/week, majority males, had more composite microvascular complication and less composite retinopathy at baseline than Black or other adults. Figure 2 displays higher odds of composite microvascular complications, nephropathy, neuropathy among White adults than the Black and other race/ethnic adults over 8 years. However, we noticed higher odds of composite retinopathy outcome among Black and other adults than the White adults. Due to the low prevalence of retinopathy, we had limited time points of recordings by race/ethnicity groups.

**Table 2:** Comparison of racial/ethnic groups based on ACCORD data (2003–2009; US and Canada; *N* = 6326)

Variable	*N*	White (*N* = 4071)^*1*^	Black (*N* = 1099)^*1*^	Other (*N* = 1156)^*1*^	*P* value^*2*^
Age	6326	62 (58, 67)	61 (57, 66)	61 (57, 66)	< 0.001
Diabetes years	6326	9 (5, 14)	10 (5, 15)	10 (5, 15)	0.004
Alcohol (drinks per week)	6326	0 (0, 1)	0 (0, 0)	0 (0, 0)	< 0.001
Sex	6326				< 0.001
Male		2690 (66%)	573 (52%)	695 (60%)	
Female		1381 (34%)	526 (48%)	461 (40%)	
Education	6326				< 0.001
≤ High school graduate (or GED)		1396 (34%)	548 (50%)	552 (48%)	
≥ Some college or technical school		2675 (66%)	551 (50%)	604 (52%)	
ACCORD intervention arm	6326				< 0.001
Standard glycemia/BP trial		929 (23%)	334 (30%)	253 (22%)	
Intensive glycemia/BP trial		901 (22%)	295 (27%)	257 (22%)	
Standard glycemia/lipid trial		1127 (28%)	236 (21%)	326 (28%)	
Intensive glycemia/lipid trial		1114 (27%)	234 (21%)	320 (28%)	
Composite microvascular complications	6326				< 0.001
No		1964 (48%)	615 (56%)	647 (56%)	
Yes		2107 (52%)	484 (44%)	509 (44%)	
Macroalbuminuria (> 300 mg/dl)	6326				0.015
No		3852 (95%)	1021 (93%)	1,072 (93%)	
Yes		219 (5.4%)	78 (7.1%)	84 (7.3%)	
MNSI	6326				< 0.001
No		2179 (54%)	708 (64%)	776 (67%)	
Yes		1892 (46%)	391 (36%)	380 (33%)	
Vibration perception	6326				< 0.001
No		2221 (55%)	774 (70%)	838 (72%)	
Yes		1850 (45%)	325 (30%)	318 (28%)	
Ankle reflexes	6326				< 0.001
No		2315 (57%)	704 (64%)	690 (60%)	
Yes		1756 (43%)	395 (36%)	466 (40%)	
Sensations from 10 g filament	6326				< 0.001
No		3229 (79%)	930 (85%)	1,008 (87%)	
Yes		842 (21%)	169 (15%)	148 (13%)	
Composite neuropathy	6326				< 0.001
No		1313 (32%)	488 (44%)	541 (47%)	
Yes		2758 (68%)	611 (56%)	615 (53%)	
Visual acuity (≤ 70 ETDRS)	6310				< 0.001
No		2821 (69%)	662 (60%)	664 (57%)	
Yes		1250 (31%)	437 (40%)	492 (43%)	
Vision loss	6326				< 0.001
No		3613 (89%)	931 (85%)	931 (81%)	
Yes		458 (11%)	168 (15%)	225 (19%)	
H/o retinopathy	6,326				
No		3700 (91%)	933 (85%)	1,017 (88%)	< 0.001
Yes		371 (9.1%)	166 (15%)	139 (12%)	
Composite retinopathy	6,326				< 0.001
No		2526 (62%)	566 (52%)	523 (45%)	
Yes		1545 (38%)	533 (48%)	633 (55%)	

^1^Median (IQR); *n* (%)

^2^Kruskal–Wallis rank sum test; Pearson’s chi squared test; Fisher’s exact test

Other race = Hispanic/Latino, American Indian/Alaska Native, First Nation (Aboriginal Canadian), Asian, Native Hawaiian/Other, Pacific Islander, French Canadian, other

*GED* General equivalency diploma, *ETDRS* Early Treatment Diabetic Retinopathy Study, *h/o* history of

Note: Other nephropathy measures were not described due to their absence at the baseline

### Mixed-Effects Models

At baseline, there was significant racial/ethnic difference of risks for the composite microvascular outcome, composite neuropathy, composite retinopathy, and vision loss even after adjusting for covariate like observed baseline outcome, diabetes year, alcohol consumption, sex, education, visit (years), and intervention arms (all *p*’s < 0.001, Table 3). The trajectory of the composite microvascular outcome, composite neuropathy, composite retinopathy, and vision loss also differed significantly by race/ethnicity after adjusting for covariates studied (all *p*’s < 0.001). There was no significant racial/ethnic difference at baseline (*p* = 0.65) or with increase in time (*p* = 0.33) for composite nephropathy outcome. Area under the curve for our models ranged from 0.91 to 0.95.

**Table 3:** Mixed method models stratified by race/ethnicity, for baseline and visit, ACCORD data (2003–2009; US and Canada; *N* = 6326)

*Outcome*	*White, OR (95% CI)*	*Black, OR (95% CI)*	*Other, OR (95% CI)*	*Overall* *P value*	*B vs. W vs. O*
*Composite microvascular complications*					
(Intercept)†	0.27 (0.22–0.34)	0.22 (0.15–0.34)	0.19 (0.12–0.30)	< 0.001	
Visit‡	1.11 (1.09–1.13)	1.06 (1.03–1.09)	0.95 (0.93–0.99)	< 0.001	B ≠ W, O ≠ W, B ≠ O
*Composite nephropathy*					
(Intercept)	0.09 (0.07–0.13)	0.10 (0.06–0.18)	0.09 (0.05–0.16)	0.648	
Visit	1.19 (1.17–1.21)	1.16 (1.12–1.20)	1.17 (1.13–1.21)	0.329	
*Composite neuropathy*					
(Intercept)	0.82 (0.63–1.06)	0.44 (0.29–0.67)	0.19 (0.11–0.32)	< 0.001	
Visit	1.09 (1.07–1.11)	1.03 (1.00–1.06)	0.97 (0.94–1.00)	< 0.001	B ≠ W, O ≠ W, B ≠ O
*Composite retinopathy*					
(Intercept)	0.99 (0.80–1.24)	1.18 (0.76–1.82)	1.72 (1.13–2.63)	< 0.001	
Visit	0.91 (0.90–0.93)	0.93 (0.90–0.97)	0.84 (0.81–0.87)	< 0.001	O ≠ W, B ≠ O
*Vision loss*					
(Intercept)	0.01 (0.01–0.02)	0 (0.00–0.01)	0.03 (0.01–0.06)	< 0.001	
Visit	1.07 (1.05–1.10)	1.1 (1.04–1.16)	0.80 (0.76–0.85)	< 0.001	O ≠ W, B ≠ O

^†^Odds for intercept

^‡^Odds ratios for visit

Odds ratios are based on models adjusted for baseline outcome, sex, diabetes years, education, alcohol, visit (years), and ACCORD intervention arm

*B* Black, *W* White, *O* Other race/ethnicity

*B ≠ W* Change of outcome in Black adults is significantly different from change of outcome in White adults over years

### Composite Microvascular Complications

At baseline, the odds of composite microvascular outcome were 0.27, 0.22, and 0.19 for the White, Black, and Other race adults after adjusting for covariates such as baseline outcome, diabetes year, alcohol consumption, sex, education, visit, and intervention arms. With increase in each year, the odds ratios of composite microvascular outcome increased to 1.11, 1.06, and 0.95 for White, Black, and Other race adults, adjusting for other covariates. In other words, for example, the odds of microvascular outcome among White adults in a given year were 1.11 times those of the previous year. The change of outcome in Black, White, and Other race adults was significantly different from each other (B ≠ W, O ≠ W, B ≠ O).

### Composite Nephropathy

At baseline, the odds of composite nephropathy outcome were 0.09, 0.10, and 0.09 for the White, Black, and Other race adults after adjusting for covariates. With increase in each year, the odds ratios of composite nephropathy outcome were 1.19, 1.16, and 1.17 for White, Black, and Other race adults, adjusting for other covariates.

### Composite Neuropathy

At baseline, the odds of composite neuropathy outcome were 0.82, 0.44, and 0.19 for the White, Black, and Other race adults after adjusting for covariates. With increase in each year, the odds ratios of composite neuropathy outcome were 1.09, 1.03, and 0.97 for White, Black, and Other race adults, adjusting for other covariates. In other words, for example, the odds of composite neuropathy among White adults in a given year were 1.09 times those of the previous year. The change of outcome in Black, White, and Other race adults was significantly different from each other (B ≠ W, O ≠ W, B ≠ O).

### Composite Retinopathy

At baseline, the odds of composite retinopathy outcome were 0.99, 1.18, and 1.72 for the White, Black, and Other race adults after adjusting for covariates. With increase in each year, the odds ratios of composite retinopathy outcome were 0.91, 0.93, and 0.84 for White, Black, and Other race adults, adjusting for other covariates. In other words, for example, the odds of composite retinopathy among White adults in a given year were 0.91 times those of the previous year. The change of outcome significantly differed between other vs. White adults, and Black vs. Other race adults (O ≠ W, B ≠ O).

### Vision Loss

At baseline, the odds of vision loss were 0.01, 0.00, and 0.03 for the White, Black, and Other race adults after adjusting for covariates. With increase in each year, the odds ratios of composite retinopathy outcome were 1.07, 1.10, and 0.80 for White, Black, and Other race adults, adjusting for other covariates. In other words, for example, the odds of vision loss among White adults in a given year were 1.07 times those of the previous year. The change of outcome in Other race adults significantly differed from change of outcome in White and Black adults (O ≠ W, B ≠ O).

## Discussion

We aimed to study differences in the longitudinal trajectories of microvascular complication risk across racial/ethnic groups among a large and diverse sample of adults. Findings from our analysis demonstrated that our hypothesis was met in part. There were significant racial/ethnic differences at baseline and over the years for all microvascular complications except nephropathy.

To our knowledge, our study addressed the gap in literature regarding the presence of racial/ethnic disparity among T2DM microvascular trajectories. Overall, we found the trajectories of composite microvascular complications showed lower risk for other race/ethnic adults than both Black and White adults over years. It was partially supported by a DPPOS study showing lower insulin sensitivity among Black compared to any other race, over 2 years [12]. Rational for such findings could be multifactorial. For instance, individual factors like genetic predisposition and medication adherence, socioeconomic factors like income and social support, and lifestyle factors like diet and cultural activities that our model could not control for. The predominance of Hispanic adults in other race group (35%) might help partially explain Hispanic paradox in diabetes, whereby Hispanic adults experience longer life expectancy despite having higher rates of diabetes and mortality [19]. However, due to the combination of several minority race/ethnicities within other race/ethnicity group, it is challenging to explain the reason for this better trajectory. Additionally, clinical trial design of this secondary analyses ensured access to standardized care to all race/ethnic groups including other race/ethnic adults. Therefore, other race/ethnicities may respond very well given an ensured access to care [11]. Emerging data suggesting subphenotyping of type 2 diabetes based on clinical characteristics or genetic clusters can help explain difference risk of complication across diverse racial/ethnic groups [20, 21]. Previous longitudinal study demonstrated association between beneficial effect of T2DM remission on lower risk of microvascular complications [22]. Another study found characteristics of T2DM population with worse HbA1c trajectories having younger, longer diabetes years and higher HbA1c levels [23]. However, studies regarding microvascular trajectories across race/ethnicities were scarce.

It is critical to discuss differences in complication risk among race/ethnicity because of the ambiguous nature of findings presented in the literature [3, 5, 9, 24]. In our study, the baseline prevalence of composite microvascular complications, neuropathy, and nephropathy were highest among White adults, followed by Black and other race/ethnic adults. Our baseline nephropathy findings did align with An et al., demonstrating a higher incidence of chronic kidney disease among non-Hispanic White adults compared to non-Hispanic Black adults [3]. On the contrary, previous studies indicated that the unadjusted incidence, or prevalence of microvascular complications and neuropathy outcomes were higher in racial/ethnic minorities such as Black or other race/ethnicity adults compared to White adults [5, 9, 24]. Such a contradictory finding may be explained by socio-demographic differences between White and Black adults within our sample. White adults had a significantly higher proportion of males, and a higher baseline prevalence of composite microvascular complications and neuropathy. Since these factors have been independently linked to complication risk, this may explain the higher odds observed among White adults in our study sample [25].

### Adjusted Vs. Unadjusted Analyses

Our findings from adjusted analysis were consistent with some studies but differed from others [5, 26]. Our adjusted analysis also showed that the adjusted odds ratio (aOR) of microvascular complications and neuropathy were highest for White adults, followed by Black adults and Other races adults. Our aOR of neuropathy were similar to an aOR of neuropathy in another longitudinal study [26]. Therefore, the racial/ethnic differences found in the unadjusted analysis of several contradicting studies might not be attributed to adults being Black or other races/ethnicities but could be due to confounding characteristics, such as education [5, 9, 24]. Therefore, we did not observe higher microvascular risks once we adjusted for such characteristics among minority racial/ethnic groups. Like previous studies, it is possible that minority racial/ethnic groups have higher risk of microvascular complications [5, 26]. Additional studies are needed to demonstrate the contrasting results between adjusted and unadjusted analyses, specifically investigating structural racism leading to inequal access to education, food, and health services [27].

### Nephropathy

Our findings for nephropathy outcomes were also comparable to other studies [11, 28]. Similar to a previous ACCORD study, our sample showed no significant difference in risk of nephropathy complications across race/ethnicity [11]. Additionally, our finding of no difference in nephropathy is similar to findings from Hamman et al., which also found no difference between non-Hispanic White and Hispanic White adults [28]. The paradox between nonsignificant racial/ethnic differences for nephropathy compared to significant differences for other microvascular complications is noteworthy. Although, such nonsignificance could be due to lower prevalence of nephropathy compared to other microvascular complications in this study, future studies with higher nephropathy prevalences are needed to verify such a paradox.

### Retinopathy

In our study, the aOR for composite retinopathy at baseline was highest for the Other race group followed by Black adults and then White adults, which partly support findings from a prior study that reported higher retinopathy among African Americans compared to non-Hispanic White adults [7]. Our retinopathy finding may contradict Hamman et al. who found higher retinopathy prevalence in non-Hispanic White adults versus Hispanic White adults [28]. Although Other race/ethnicity in our study mainly comprised Hispanics, we were unable to distinguish Hispanic adults by racial identity (White vs. Black). Such diversity and different comparison groups may explain the contradicting nature of the findings. In addition, the difference in outcome measurements may explain difference in findings; Hamman et al. measured retinopathy using stereo fundus photographs; whereas in the ACCORD study, it was measured using visual acuity, vision loss, and history of retinopathy. Lastly, the prevalence of retinopathy in Hamman et al. was only adjusted for diabetes duration. In contrast, our study adjusted for several factors beyond diabetes duration, which could further explain the differences in findings.

### Progression and Severity of Complications

Less is known about the progression of microvascular complications over the years. Previous studies focused on T2DM progression [9, 12]. By investigating T2DM microvascular complications, our findings partially support that T2DM progression occurs faster among Black adults [9, 12]. Composite microvascular complications, composite neuropathy, and vision loss as a measure of retinopathy worsened more for Black and White adults over the years. In contrast, Other adults, mainly comprised of Hispanic adults (35% of other race), showed yearly improving trajectories for such outcomes. Despite having higher odds of composite retinopathy at baseline, over time, the odds of retinopathy decreased steeply for Other adults. Odds ratios of composite retinopathy for White and Black adults also decreased over the years. On the other hand, when retinopathy was measured by vision loss, the odds ratios did not decrease over the years for White and Black adults. Vision loss is a severe form of retinopathy compared to our measurement of composite retinopathy. This explains that the racial/ethnic disparity may also depend on the severity of microvascular complications which was similarly found by Hamman et al. [28]. Future researchers should consider severity of microvascular complications to avoid under-recognition of racial/ethnic disparities.

### Strengths and Limitations

Our study has strengths and limitations. The data source was a strength because, despite being relatively older, it provided detailed information on demographic and microvascular measures, which permitted robust longitudinal analyses. However, given differences in the availability of microvascular measures at different time points, not all the analyses performed featured the same time points and sample sizes. Nevertheless, we do not believe this affected our analysis because the goal was to study the initial outcome and its changes over time with at least three time points. The ACCORD study sample was limited to older adults, so we could not study the younger population. Therefore, findings from our study should not be interpreted for younger adults with T2DM. During the analytic phase, we reduced misclassification bias by using standard definitions for the outcomes to be consistent with previous studies including primary ACCORD study.

Another key limitation was our inability to control an exhaustive list of covariates and relevant factors such as annual income, health insurance status, the burden of comorbidities, and geographic access to diabetes education and management programs. However, due to the randomization of study participants, we believe most of the unknown confounders were already controlled in the design phase. The benefit of having data on factors such as health insurance status and access to care is that it allows us to explore the importance of underlying structural factors related to microvascular complication risk [14, 15]. Race/ethnicity as a variable itself does not represent structural racism and its historical effect on racial/ethnic health disparities. Moving forward, studies should examine upstream determinants and their impact or risk of T2DM and related complications [13, 14]. Finally, there might be heterogeneity within other race/ethnic groups due to a combination of various race/ethnicities. Grouping diverse identities within other race/ethnic group may obscure subgroup specific risks.

### Sensitivity Analysis

Sensitivity analysis was performed to investigate any possibility of bias resulting in our subsample due to the exclusion of participants (Supplemental Table 1). Dropout rates were higher among women, older adults, less educated, Black, and Hispanic groups. Such an attrition bias could underestimate the burden of disease of these groups. Our results suggest further studies should implement specific strategies to retain these specific groups. The specific dropouts in our study might have affected our study results in two ways. Firstly, Black and Hispanic participants who were retained in our sample could be those with higher education and income. If this is the case, the true effect might be more exaggerated than our findings suggest when more marginalized individuals are included. Secondly, the statistically significant difference across included vs. excluded samples might have been driven more by statistical power than the true clinical difference (see Supplemental Table 1). Lastly, the exclusion of participants with limited data might have introduced selection bias that disproportionately affected racial/ethnic minority groups such as Black, Hispanic, and Asian adults. However, ACCORD study made significant efforts to recruit racial/ethnic minorities. Future randomized trial studies should recruit racial/ethnic minorities with consideration of differential selection and dropout rate.

### Implications

This study adds to the current literature on racial/ethnic disparities among T2DM microvascular complications. Our findings may be helpful for researchers, practitioners, public health professionals, policymakers, and organizations that work with populations with T2DM. The limitations of our study and the results from our sensitivity analyses could help future studies in retaining racial/ethnic minority groups and powering their analyses to consider these groups. It also guides them to understand some of the possible rationale behind mixed findings. For instance, clinical trial designs can show minority race/ethnic groups to have lower risk. However, it is vital to understand the significance of study design and demographic of minority race/ethnicity. Such results might have resulted because clinical trial designs ensure that everyone has access to health care. Additionally, composition of racial/ethnic minority groups could be from higher income and education demographic. Future clinical and policy intervention development should consider racial/ethnic disparities to deliver equitable care and improve healthcare quality.

## Conclusion

In conclusion, we found that most microvascular complications, except nephropathy, significantly differed across race/ethnicity. Black and White adults not only have worse outcomes to start with but also worse progression over years compared to other race/ethnicity adults. For the other race/ethnic group, most microvascular complications had significantly lower risk trajectories over years except nephropathy. Our findings highlight the need to include a diverse population of racial and ethnic groups that are often not examined in studies of T2DM health outcomes. Diversifying study populations can be challenging. Therefore, the number of subgroup-specific studies (e.g., Asians, Hispanics, American Indians/Alaskan Natives) should increase in the near future to validate our findings. Future studies should also explore behavioral, social, and environmental drivers for racial/ethnic disparities in T2DM microvascular complications among adults.

## Supplementary Information

Below is the link to the electronic supplementary material.

Supplementary file1 (DOCX 17 KB)

## Data Availability

The data used in this study are from the Action to Control Cardiovascular Risk in Diabetes (ACCORD) trial, which is available through the National Heart, Lung, and Blood Institute (NHLBI) Biologic Specimen and Data Repository Information Coordinating Center (BioLINCC). Access to the data requires submission of a research proposal and approval from BioLINCC. The dataset can be requested at: https://biolincc.nhlbi.nih.gov/studies/accord/.
