# Supplementary material for: Risk Trajectories of Type 2 Diabetes–Related Microvascular Complications Across Racial/Ethnic Groups Among Older Adults
Source: J Racial Ethn Health Disparities. 2025 Sep 9;13(6):4807–17. doi: 10.1007/s40615-025-02625-0 (PMC13754763; doi:10.1007/s40615-025-02625-0)
Supplement: Supplementary file 1 [file 40615_2025_2625_MOESM1_ESM.docx]

### Supplemental Table 1: Sensitivity analysis of sample included vs. excluded, ACCORD data (2003-2009; US and Canada; N = 6,326)

| Variable | Sample excluded,  N = 3,925*^1^* | Sample included,  N = 6,326*^1^* | P-value*^2^* |
| --- | --- | --- | --- |
| Sex |  |  | 0.003 |
| Male | 2,341 (60%) | 3,958 (63%) |  |
| Female | 1,584 (40%) | 2,368 (37%) |  |
| Baseline age | 62.50 (58.10, 67.60) | 61.80 (57.50, 66.70) | <0.001 |
| ACCORD Intervention arm |  |  | 0.12 |
| Intensive Glycemia/Lipid Fibrate | 546 (14%) | 828 (13%) |  |
| Intensive Glycemia/Lipid Placebo | 543 (14%) | 840 (13%) |  |
| Intensive Glycemia/Intensive BP | 465 (12%) | 713 (11%) |  |
| Intensive Glycemia/Standard BP | 453 (12%) | 740 (12%) |  |
| Standard Glycemia/Lipid Fibrate | 553 (14%) | 838 (13%) |  |
| Standard Glycemia/Lipid Placebo | 519 (13%) | 851 (13%) |  |
| Standard Glycemia/Intensive BP | 410 (10%) | 774 (12%) |  |
| Standard Glycemia/Standard BP | 436 (11%) | 742 (12%) |  |
| History of Cardiovascular disease at baseline |  |  | <0.001 |
| No | 2,455 (63%) | 4,187 (66%) |  |
| Yes | 1,470 (37%) | 2,139 (34%) |  |
| Race |  |  | <0.001 |
| Black | 854 (22%) | 1,099 (17%) |  |
| Hispanic | 332 (8.5%) | 405 (6.4%) |  |
| Other | 417 (11%) | 751 (12%) |  |
| White | 2,322 (59%) | 4,071 (64%) |  |
| Education |  |  | <0.001 |
| Less than high school graduate | 698 (18%) | 823 (13%) |  |
| High school graduate (or GED) | 1,031 (26%) | 1,673 (26%) |  |
| Some college or technical school | 1,234 (31%) | 2,123 (34%) |  |
| College graduate or more | 955 (24%) | 1,707 (27%) |  |
| *^1^* n (%); Median (IQR)  ^2^ Pearson’s Chi-squared test; Wilcoxon rank sum test  GED=General equivalency diploma | | | |
